# Aptamers Targeting Cardiac Biomarkers as an Analytical Tool for the Diagnostics of Cardiovascular Diseases: A Review

**DOI:** 10.3390/biomedicines10051085

**Published:** 2022-05-06

**Authors:** Natalia Komarova, Olga Panova, Alexey Titov, Alexander Kuznetsov

**Affiliations:** Scientific-Manufacturing Complex Technological Centre, 1–7 Shokin Square, Zelenograd, 124498 Moscow, Russia; panovaolya691@gmail.com (O.P.); alextitov2187@mail.ru (A.T.); kae@tcen.ru (A.K.)

**Keywords:** cardiovascular disease, cardiac biomarkers, aptamer, detection, biosensor, diagnostics

## Abstract

The detection of cardiac biomarkers is used for diagnostics, prognostics, and the risk assessment of cardiovascular diseases. The analysis of cardiac biomarkers is routinely performed with high-sensitivity immunological assays. Aptamers offer an attractive alternative to antibodies for analytical applications but, to date, are not widely practically implemented in diagnostics and medicinal research. This review summarizes the information on the most common cardiac biomarkers and the current state of aptamer research regarding these biomarkers. Aptamers as an analytical tool are well established for troponin I, troponin T, myoglobin, and C-reactive protein. For the rest of the considered cardiac biomarkers, the isolation of novel aptamers or more detailed characterization of the known aptamers are required. More attention should be addressed to the development of dual-aptamer sandwich detection assays and to the studies of aptamer sensing in alternative biological fluids. The universalization of aptamer-based biomarker detection platforms and the integration of aptamer-based sensing to clinical studies are demanded for the practical implementation of aptamers to routine diagnostics. Nevertheless, the wide usage of aptamers for the diagnostics of cardiovascular diseases is promising for the future, with respect to both point-of-care and laboratory testing.

## 1. Introduction

**Figure 1:**
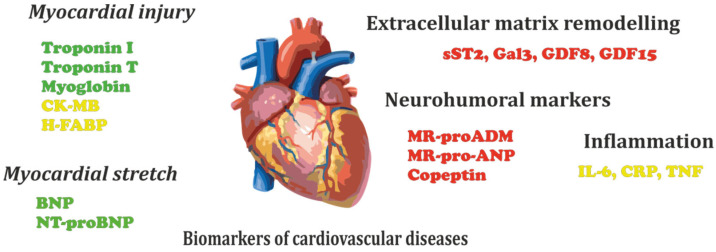
Classification of cardiac biomarkers (adopted from reference [3]). CK-MB—creatine kinase; BNP—B-type natriuretic peptide; NT-proBNP—N-terminal pro-B-type natriuretic peptide; MR-proADM—mid-regional proadrenomedullin; MR-proANP—mid-regional pro-atrial natriuretic peptide; IL-6—interleukin-6; CRP—C-reactive protein; TNFα—tumor necrosis factor alpha; sST2—soluble suppression of tumorigenicity 2; Gal3—galectin-3; GDF8—growth differentiation factor 8; GDF15—growth differentiation factor 15. Aptamers are well established as an analytical tool for the biomarkers highlighted in green; aptamers are known, but not sufficient for practical analysis, for the biomarkers highlighted in yellow. No aptamers are known for the biomarkers in red.

Cardiovascular diseases (CVDs) are the most frequent cause of death worldwide. The WHO estimated the number of deaths associated with CVDs to have reached 17.9 million in 2019, about 32% of all global deaths [1]. The early detection of CVDs is required to start medical treatment as soon as possible. The diagnostics of CVDs rely on physical examinations, electrocardiography, echocardiography, and blood analysis [2]. A variety of cardiac biomarkers have been established which are useful for diagnostics, progression evaluation, outcome prognosis, and the risk assessment of CDVs. These biomarkers are classified in several categories (Figure 1). Cardiac troponin I (cTnI), cardiac troponin T (cTnT), myoglobin, and creatine kinase (CK-MB) are considered as specific markers of myocardial injury. B-type natriuretic peptide (BNP) and N-terminal pro-B-type natriuretic peptide (NT-proBNP) are the markers of myocardial stretch. Neurohumoral markers include mid-regional proadrenomedullin (MR-proADM), mid-regional pro-atrial natriuretic peptide (MR-proANP), and copeptin. Inflammatory biomarkers are also related to CDVs, with interleukin-6 (IL-6), C-reactive protein (CRP), and tumor necrosis factor alpha (TNFα) being the most notable among them. The soluble suppression of tumorigenicity 2 (sST2), galectin-3 (Gal3), growth differentiation factor 8 (GDF8), and growth differentiation factor 15 (GDF15) are the markers of extracellular matrix remodeling [3]. The speed, accessibility, and low cost are the key benefits of biomarker analysis, as it can be performed both in laboratory environments and using point-of-care portable (POC) devices [4].

To date, biomarker analysis mainly relies on immunoanalysis with special concern to laboratory diagnostics, which implies mainly sandwich antibody-based detection. As a diagnostics tool, antibodies offer high sensitivity and selectivity, but also have some drawbacks, which are relatively high price and batch-to-batch variations, the latter leading to the loss of result reproducibility [5]. To overcome these issues, aptamers are emerging as an alternative to antibodies as an analytical instrument. Aptamers are short nucleic acid ligands (DNA or RNA of 15–100 nt long) which are capable of binding to a specific target with high affinity and specificity [6]. SOMAmers (slow off-rate modified aptamers) are DNA aptamers in which nucleic bases are modified with some hydrophobic moieties [7]. These modifications expand the range of aptamer–target interactions with hydrophobic ones, which are absent in conventional DNA and RNA aptamers. This, in turn, results in higher affinities of SOMAmers to their targets [7]. Aptamers to almost any target can be obtained using an in vitro evolutionary approach called SELEX [8,9]. The structure of any aptamer is standardized by its primary sequence, which is always known for the aptamer, in contrast to antibodies. The automation of chemical oligonucleotide synthesis minimizes the probability of batch-to-batch variations. DNA and RNA oligonucleotide synthesis is available from different manufacturers for a cost of about USD 0.2–0.5 per single nucleotide. Thus, aptamers offer a low cost of chemical synthesis, very low batch-to-batch variation, ease of labeling, and regeneration ability [10,11]. An additional benefit of aptamers is the possibility to regulate their selectivity among the interfering analytes using negative and counter selection steps introduced to the selection procedure. This ability is one more advantage of aptamers in comparison to antibodies, which are suspected to exhibit cross-reactivity to homologous targets [12]. Aptamers expand strongly to the research field of POC device development, with cardiac biomarker detection systems being among them [3]. As for laboratory diagnostics, aptamer-based detection often lacks sandwich-type analysis, while this analysis has gained its popularity due to the enhanced specificity of assays granted by dual-target recognition and the ability to implement signal amplification labels to one of the paired ligands [13]. Sandwich-type analysis is applicable to both electrochemical and optical detection platforms and is useful for laboratory and POC diagnostics. A drawback of aptamers, in respect to analytical applications, is a strong impact of the surrounding conditions on aptamer folding, and thus target recognition; the binding of aptamers to targets is strongly affected by pH and buffer composition [6]. This can impede the practical detection of biomarkers from biological fluids, which is strongly important for POC devices.

This review is aimed to critically evaluate the existing aptamers to the most common CVD biomarkers as a basis for analytical detection for the purpose of practical implementation to clinical diagnostics. For this, binding characteristics and specificity of the established aptamers to the targets, as well as the ascertainment of sandwich-type aptamer assays, are analyzed. Special attention is paid to the possibility of CVD biomarker detection from whole blood, saliva, and urine as alternatives to blood serum, as these fluids are more affordable for POC formats. Aptamers have not been established for all cardiac biomarkers. Nevertheless, we include the biomarkers for which aptamers are not yet discovered to this review to provide a more exhaustive biomarker range and to demonstrate the gaps in the current aptamer research.

## 2. Cardiac Biomarkers and Their Aptamers

### 2.1. Cardiac Troponins

The troponin complex, which regulates muscle contraction, is a component of skeletal and cardiac muscle thin filaments [14,15]. Myocardial damage results in the release of troponin to blood circulation within 1–3 h of injury [14]. The peak concentration of troponin, mounting up to 100 ng/mL, is reached within 1–2 days after the infarction, and the elevated level remains for about 10 days [16]. The troponin complex is comprised of three subunits: troponin C, troponin I, and troponin T. In humans, troponins I and T exist in three isoforms each: fast and slow skeletal isoforms and a specific cardiac isoform (cTnI and cTnT) [14,15], while troponin C is identical in skeletal and cardiac tissues [15]. Due to this, cTnI and cTnT are rather sensitive and specific biomarkers for cardiac injury. Elevated levels of cTnI and cTnT in blood serum are associated with acute myocardial infarction (AMI), but can also be caused by other reasons, both cardiac and non-cardiac [14,17]. Besides blood serum, cardiac troponins can be measured in saliva and urine. Saliva levels of cardiac troponins correlate with blood serum levels and, therefore, detection from saliva can be used instead of blood analysis. Cardiac troponins in urine are associated with renal disease [14].

To date, troponins are the most specific biomarkers for myocardial injury. In clinical practice, sandwich immunological assays are dominant for the detection of troponins. Contemporary ultra-sensitive immunoassays are capable to detect as low as 1 ng/L cTnI [15]. Nevertheless, the detection of troponins is hampered by the cross-reactivity of sensitive cTnI and cTnT antibodies with skeletal isoforms of troponins, which causes the false positive results of detection [15,18]. Due to possible unspecific detection, the diagnostics cut-off value should be established for each detection method individually based on the analysis of troponin levels in a healthy control group [16,19]. Today a lot of effort is posed to develop more and more acute, sensitive and fast detection tools for cTnI and cTnT.

For cTnI, several DNA aptamers have been reported. In 2015, H. Jo et al. published the isolation of Tro4 and Tro6 aptamers binding cTnT with K*_D_* in the picomolar range [20]. The specificity of cTnI detection using Tro4 was demonstrated over cTnT, cTnC, human serum albumin (HSA), myoglobin, and B-type natriuretic peptide [20]. Tro4 has become the most popular tool for the development of numerous biosensors and detection methods [21,22,23,24,25,26,27,28]. Many sensors have also been developed using the Tro6 aptamer [29,30]. The specificity of Tro6 for cTnI has been proven using thrombin, myoglobin, IgG, BSA, and alpha-fetoprotein [30]. The combination of Tro4 and Tro6 allowed the development of sandwich assays for cTnI detection [20,31,32,33,34,35].

Dorraj et al. performed aptamer isolation resulting in four aptamer sequences (TnIApt23, TnIApt19, TnIApt18, TnIApt11) [36]. The sequences of TnIApt23 and TnIApt19 are not provided in the original paper, but a dot-blot assay for cTnI detection based on the TnIApt23 aptamer is reported beside the aptamer selection [36]. TnIApt18, the sequence of which is published, was further used by other research groups for the development of other cTnI biosensors [37,38].

In 2018, the TnI2 aptamer was isolated [39] and further applied for the ELONA-type assay of cTnI based on the combination of the aptamer and the TnI-specific antibody [40].

Cen et al. reported the selection of two more DNA aptamers for cTnI named Apt 3 and Apt 6, and their application for the ELONA-type sandwich assay for cTnI detection [41]. The developed aptamer-based assay was characterized with a dynamic range of 0.05–200 ng/mL. The detection of cTnI was not interfered with by skeletal TnI, cTnT or cTnC. This research represents a rather rare example of aptamer-based assay validation in clinical diagnostics. The aptamer-based ELONA was compared to a commercial antibody-based FICA kit for cTnI detection in the clinical diagnosis of AMI. The sensitivity and specificity of the aptamer-based ELONA were 96.46% and 93.85%, respectively, and the accuracy was 95.06% These parameters turned out to be superior to the FICA kit which is used in clinical practice [41].

The group of Tamás Mészáros developed Spiegelmers targeting cTnI [18,42]. Spiegelmers are L-DNA ligands exhibiting very strong nuclease resistance. The peptide fragments from both C and N terminal parts of cardiac troponin I serve as selection targets to enable subsequent sandwich-type detection and avoid cross-reactivity of the Spiegelmers with skeletal TnI isoforms. Four Spiegelmers, two (A4, B10) targeting the C-terminal fragment [18] and two (A6, C6) for the N-terminal fragment [42], were established. These Spiegelmers were evaluated for the development of a sandwich bead-based luminescent homogenous proximity assay (AlphaLISA) [18,43]. Interestingly, the B10 sequence has also been reported to function as a conventional DNA aptamer. Electrochemiluminescence disposable [44] and electrochemical (DPV) [45] aptasensors employing DNA B10 sequence have been reported, but no mention that it is a Spiegelmer is present in these papers.

Krasitskaya et al. reported DNA aptamer selection against cTnI which resulted in several aptamer sequences (TnAp1, TnAp2, TnAp4, TnAp5, TnAp10, TnAp12, TnAp14, TnAp2t1, TnAp2t2, TnAp2t3) [46]. Based on TnAp10 and TnAp2t3, a sandwich bioluminescent assay for cTnI detection capable to detect 0.04–3 nM of cTnI was developed [46].

A sequence of the DNA aptamer for cTnI is provided in [47]. This paper describes a SERS sensor for cTnI detection. One more cTnI aptamer sequence is available in [48]. Based on this aptamer, an electrochemiluminescent aptasensor for cTnI was developed. Additionally, two research works report aptasensors which seem to employ unique aptamers, but no sequences are provided in the publications [49,50].

The known sequences of aptamers binding to cTnI and other cardiac biomarkers discussed in this review are summarized in Table 1.The existing cTnI aptasensors are extensively reviewed in special papers [51,52]. The large variety of detection platforms for the aptasensing of troponins is available, including both optical assays (luminescent, fluorescent, SPR, SERS, colorimetric) and electrochemical biosensors (DPV, CV, SWV, EIS, FET). Most of the developed aptasensors display excellent sensing characteristics. The established aptamers are applicable for the development of both laboratory assays with instrumental detection [29,37,40,42] and POC portable devices [35,38], can be combined to benefit with sandwich-type analysis [32,33,34,35,42,53], and have been shown to detect cTnI in saliva, urine and whole blood, as well as blood serum [21,23,51]. Surely, aptamers can serve for cTnI detection in clinical practice. Among the varieties of aptamer binding, cTnI, Tro4 and Tro6 display the best performance due to the lowest K*_D_* values, well demonstrated specificity to cTnI, applicability for the detection from saliva and serum, and ability to function in dual-aptamer sandwich assays. Moreover, Apt 3 and Apt 6 are viable for practical usage, as this aptamer pair display good LOD and vast dynamic range for cTnI detection, and have been evaluated in clinical research.

The specificity of cTnT for AMI detection is lower compared to cTnI [54,55]. However, cTnT is believed to be the most stable AMI biomarker [54], and due to this displays higher accuracy in late diagnostics [55]. cTnT levels correlate with mortality in patients, making it a disease outcome predictor [55].

cTnT concentration in saliva correlates with its concentration in blood serum [56], and cTnT detection from saliva can be an alternative for blood testing.

Several aptamers are also available for Troponin T. The Tro4 aptamer, which was initially developed for cTnI [20], has been reported to detect cTnT electrochemically [57,58]. The selectivity test was performed for both aptasensors, but cTnI was not included in the interferents set. At the same time, the specificity of the Tro4-based aptasensor against cTnT was addressed in other papers, and the reported aptasensors for cTnI detection exhibited no response to cTnT [20,24,25,28]. So far, the data on the specificity of Tro4 for cTnT is confusing. This places special emphasis on the necessity of the specificity assessment of the aptamers and aptamer-based detection methods to the desired target and to appropriate cross-target choice.

Two more DNA sequences (Apt1 and Apt2) have been found to work for the ELONA sandwich assay of cTnT [59]. The LOD and detection range of this assay were not sufficient for the real detection of cTnT, but demonstrated the benefit of good analyte recovery from undiluted serum [59]. The sensing parameters of the sandwich assay based on Apt1 and Apt2 can be improved with the application of other biosensor design. Apt1 was applied to detect cTnT electrochemically, and the sensor demonstrated an LOD of 1.7 pg/mL from 10-fold diluted serum, which is enough for practical usage, and showed no response to cTnI and myoglobin [60]. A DNA aptamer binding to Troponin T (AraHH001) was discovered during cell-SELEX against mouse tumor endothelial cells [61,62]. It also binds human tumor endothelial cells, and this research revealed that cTnT can serve as a cancer marker in human [62]. AraHH001 was used for electrochemical cTnT detection as a part of multiple biomarker detection panel [63]. Besides, two papers report cTnT FET-based aptasensors with no indication of the employed aptamer sequences [64,65]. The amount of both established aptamer sequences and the developed aptasensors for cTnT is less than for cTnI. Nevertheless, the existing aptamers provide a sufficient basis for the implementation of aptamer-based cTnT detection in clinical practice.

### 2.2. Myoglobin

Myoglobin is a rather small protein with a molecular weight of 16.8 kDa whose function is the binding, storage and transport of oxygen. It is present in skeletal and cardiac muscles. The normal blood level of myoglobin is reported to be 6–85 ng/mL [54]. Upon the injury of muscle cells, which happens under AMI, it is released in the blood and reaches the levels of 70–200 ng/mL, which is the diagnostics cut-off value for myoglobin [66]. Myoglobin is the earliest AMI biomarker, as its level in blood increases within 1 h after infarction, reaches its maximum at 4–12 h, and returns to normal within 24 h [54,67]. Due to the presence in skeletal muscles, myoglobin is not absolutely specific for cardiac diseases, but it exhibits a high negative predictive value [68]. Myoglobin can be detected in saliva [69,70,71] for AMI diagnostics and in urine, though in urine it serves as a renal and muscle disease marker, but not cardiac [72,73,74].

A successful aptamer isolation against myoglobin was reported in 2014 [75]. Three aptamer sequences are provided in the respective paper (Myo40-7-27, Myo40-7-69, Myo40-7-34) and, among them, Myo40-7-27 exhibited the lowest K*_D_* of 4.93 nM. Myo40-7-27 has generated dozens of biosensors of different types using electrochemical and optical platforms [76]. Myo40-7-27 can be combined with myoglobin-specific antibodies to perform the assay in a sandwich format [77,78]. Besides, the established splitting of this aptamer enables the usage of donor–acceptor proximity-based detection, which is usually a benefit of sandwich-type assays [79].

Three more DNA aptamers for myoglobin are reported, but these sequences are much less involved in aptasensor development. One of them is a DNA aptamer with a length of 72 nt produced by OTC Biotech [80]. The aptamer was applied to develop a DPV-based electrochemical sensor with a 2.1 pg/mL limit of detection (LOD) from 10-fold diluted human serum. Another aptamer is referred to as ST1, but its sequence is not available [81]. ST1 served for the detection of myoglobin as part of an electrochemical sensor with an LOD of 0.34 ng/mL [81] and the SERS sensor detecting as low as 10 fg/mL [82]. Finally, the isolation of the 78 nt long DNA aptamer, characterized with picomolar K*_D_* value, was reported [83]. The aptamer was applied for the electrochemical detection of myoglobin in serum samples with an LOD of 0.524 pg/mL. The sensor provided no response for hemoglobin and BSA [83].

An extensive recent review of aptamer-based nanosensors for myoglobin is available [76]. In brief, the variety of myoglobin aptasensors was developed using different detection platforms. LODs and detection ranges efficiently covered the required clinical values. No two-side sandwich assay based completely on the aptamers for myoglobin has been reported to date, though this approach could be very useful for developing myoglobin assays in a popular ELISA-type format. Most of the sensors are applied for the detection from serum samples, while detection from saliva [84] and urine [84,85] is performed rarely. No aptasensing of myoglobin in whole blood has been described [76]. The described aptamers for myoglobin have equal chances to be employed for the clinical detection of this biomarker. For this, the evaluation of sandwich aptamer pairs and more efforts for the development of the sensors for the detection from saliva and urine are required.

### 2.3. Creatine Kinase

Creatine phosphokinase (CK, EC 2.7.3.2) is present in humans in three isoenzymes—BB, MM and MB—the names of which originate from the various combination of the M (i.e., muscle) and B (i.e., brain) isoforms. A significant concentration of CK-MB isoenzyme is found almost exclusively in the myocardium, and the appearance of elevated CK-MB levels in serum is highly specific and sensitive for myocardial cell wall injury. Normal reference values for serum CK-MB range from 3 to 5% (percentage of total CK activity) or 5 to 25 IU/L. The peak level of CK-MB activity is reached 3–8 h after infarction, ranges from 15 to 30% of total CK activity, and stays elevated for 24–48 h [86]. Despite the fact that the introduction of assays for troponins T and I has allowed the slightly earlier diagnosis of acute coronary syndrome (ACS) and improved the sensitivity over CK-MB [87,88], the measurement of CK and CK-MB is still popular in clinical diagnostics [89]. The evaluation of CK-MB in serum allows the determination of infarct size, and the infarct size is associated with short- and long-term cardiovascular complications such as cardiac death, reinfarction, congestive heart failure (CHF), stroke and unstable angina requiring hospitalization [90]. The presence of CK in saliva could confer a quick, less invasive investigation at lower cost. Mirzaii-Dizgah et al. investigate the serum and unstimulated whole saliva of AMI patients and healthy participants to compare the ratio of CK-MB/whole CK [91] and reveal the higher levels of CK-MB in the AMI group. The same correlations were reported in [92], but these correlations do not allow selective diagnosis as serum measurements [93] and additional study should be performed.

Nowadays, the CK-MB level is usually determined by the ELISA based on the sandwich principle with a mono-clonal antibody specific for the MB isoenzyme [94] or by the activity measurements of the B-subunit after the immunoinhibition of M-subunit activity [95].

Two DNA aptamers (c.Apt.21 and c.Apt.30) with high specificity and affinity to CK-MB are reported [96]. By using fluorescent microspheres and c.Apt.30, an aptamer-based lateral flow assay was developed and tested on artificial serum samples. The LOD for CK-MB was found to be as low as 0.63 ng·mL^−1^, and the assay worked in the range of 0.005–2 μg·mL^−1^ CK-MB concentration. This makes it promising that a developed assay can be used clinical diagnostics [96]. In another paper, c.Apt.21 was used to develop a microfluidic chip with DNA hydrogel for the detection of CK-MB. The obtained signal was linearly correlated with the logarithm of CK-MB concentration in the range of 0.01–750 nM. In this approach, a cell phone was used to perform a quantitative readout of reaction results with an LOD of 0.027 nM [97].

### 2.4. Heart-Type Fatty Acid-Binding Protein

Heart-type fatty acid-binding protein (HFABP) is a small protein with a molecular weight of 15 kDa [98]. HFABP is assumed to contribute to the uptake, intracellular metabolism, and transport of long-chain fatty acids [99]. This protein is a cardiac-specific form of fatty-acid binding proteins and is abundant in cardiomyocytes [99]. HFABP increases in the blood within 30–90 min of cardiac injury [3,100], reaches its maximum at 6–8 h, and returns to baseline after 24–36 h [4,100]. Due to its fast release to blood, HFABP can serve as an early biomarker of ACS [3,101], being superior to myoglobin for AMI detection due to its faster release and the specificity of the cardiac form [4]. HFABP can be used to detect recurrent myocardial infarction [4] and to evaluate the risk of adverse cardiac events [102]. HFABP has also been detected in the urine of patients with AMI [103]. No information can be derived from the scientific literature on the presence of HFABP in saliva. Normal HFABP concentration in blood is typically less than 4.3 ng/mL [102]. The diagnostics cut-off value is not uniformly established, and ranges from 4 to 5.7 ng/mL [3,102]. High HFABP levels (9.3–79.0 ng/mL) are reported in sepsis patients [3,104]. ELISA is a common method for HFABP detection in practice [105].

Only one paper concerning aptamer development against HFABP has been published [101]. Two DNA aptamers (N13 and N53) with distinct binding sites were isolated with K*_D_* values of 74.3 and 334 nM, respectively. An application of these aptamers for HFABP detection using an AuNP aggregation-based colorimetric assay provided an LOD of 54 ng/mL, which is insufficient for practical usage.

So far, the future direction of aptamer research for HFABP is the isolation of novel HFABP-specific aptamers and the development of new detection methods, including sandwich assays.

### 2.5. B-Type Natriuretic Peptide and N-Terminal Pro-B-Type Natriuretic Peptide

B-type natriuretic peptide (BNP) is released in response to myocardial stretching [106,107]. Cardiomyocytes produce a proBNP, a 108-amino-acid peptide which is then enzymatically cleaved at the 76–77 position to form N-terminal proBNP (NT-proBNP, 1–76 amino acids of pro-BNP) and BNP (77–108 amino acids of proBNP). BNP contains a 17-amino-acid ring closed with a disulfide bond [107]. BNP is physiologically active, and NT-pro-BNP does not display biological activity [2,4]. BNP dilates blood vessels, decreases vascular resistance, increases stroke volume and renal sodium secretion, and increases urine production, which results in a decrease in blood volume and hence blood pressure [108]. BNP and NT-proBNP serve as biomarkers of HF and ACS and have prognostic utility in HF and coronary heart disease [109]. Regardless, these peptides are not considered to be exclusively cardiac specific [110]. Though BNP and NT-proBNP are released in equimolar amounts, BNP has a shorter lifetime in blood, which is 20 min for BNP and 1–2 h for NT-proBNP [111]. A normal value of BNP in the blood is about 20 pg/mL, and the diagnostic cut-off level is 0.1 ng/mL [2,4]. The cut-off value for NT-pro-BNP is 0.3 ng/mL [110]. So far, the detection of BNP is more difficult compared to NT-proBNP, due to its low levels and short in vivo and in vitro lifetime [110,111]. BNP is degraded by unspecific proteases to shorter peptide fragments with no biological activity, which are still immunoreactive, and this additionally hampers the correct detection of BNP [110]. Both BNP and NT-proBNP can be detected from saliva [112,113,114], which is acceptable for HF diagnostics and monitoring [114].

The first aptamer binding to BNP referred to as 8–12 was published in 2009 [115]. No K*_D_* is provided for this 55 mer DNA aptamer, as it was selected among other candidate sequences based on an ELISA-type end-point screening assay [115]. Nevertheless, this aptamer encouraged the development of various biosensors based on different detection platforms including SPR [115,116,117], FRET [118], and DPV [119]. The mentioned SPR and DPV sensors employed BNP-specific antibodies besides the BNP aptamer to build a sandwich-type binding of the target. The dynamic range of the aptasensors fits the biological levels of BNP. The detection of BNP with these sensors was performed in spiked serum samples [119] and in the diluted blood [115].

To develop a sandwich assay of BNP fully based on aptamers, an aptamer isolation experiment was performed by Bruno et al. [120]. The candidate aptamer sequences were screened for binding ability using ELONA, and four oligonucleotides, which provided maximum binding signal (2F, 6R, 14bF, 25cF), were selected for subsequent evaluation in an aptamer-magnetic bead capture electrochemiluminescence sandwich assay of BNP. Maximum response was obtained for the combination 25c-2F, but the cross-reactivity of these oligonucleotides was insufficient with notable response to BSA, CRP, and IL-6 [120].

Another aptamer selection against BNP was reported in 2014 by Wang et al. [121]. The selection resulted in several potent DNA aptamer candidates named A8, A10, A11, A14-1, and A14-5 which displayed target binding in a fluorescence binding assay. Among them, A10 oligonucleotide with the lowest K*_D_* of 12 nM was established as a BNP aptamer, its specificity was proven over BSA and Ovalbumin, and no matrix effect on the binding was detected in the serum [121]. The A10 aptamer to BNP was applied for the development of the photoelectrochemical (PEC) sensor with an LOD of 0.14 pg/mL and a linear range 1 pg/mL–0.1 mg/mL. The aptasensor was applied for the detection of BNP in serum [122]. Another sensor engaging the A10 aptamer is a DPV-based electrochemical biosensor capable of detecting 1 pg/mL–1 mg/mL BNP in spiked serum [111]. Notably, in this research, no BNP was detected in the clinical samples, either with the aptasensor or with the laboratory immunoassay, while NT-proBNP was determined with the laboratory method in the same samples. This demonstrates the influence of the very fast degradation of BNP on its assay [111].

The aptamers 8–12 [115] and A10 [121] are the most promising for future implementation to clinical diagnostics.

As for NT-proBNP, we succeeded to find a single DNA aptamer sequence named N20a which was isolated along with the selection aptamers for cTnI and fibrinogen performed by Sinha et al. using the microfluidic SELEX platform [39]. The binding of N20a with NT-proBNP was characterized with a K*_D_* of 2.89 nM determined with SPR, and the aptamer was shown to exhibit no cross-reactivity for cTnI, fibrinogen or BSA. The aptamer sequence was hidden in the original paper, but was later published in another report of the same research group [123]. N20a was applied for NT-proBNP detection in an automated microfluidic platform [123]. The detection relied on a combination of aptamer and specific antibodies in a sandwich-type assay. The LOD was established to be as low as 1.53 pg/mL. The detection of the target from spiked serum samples provided 86.3–96.9% recovery. The results of NT-proBNP detection with the developed microfluidic system were consistent with the results of the clinically involved immunoassay Cobas e411 (Roche Diagnostic) [123]. An amperometric aptasensor for NT-pro-BNP based on N20a is described. This sensor was realized in two variants, one based on the direct detection using the aptamer and one based on the aptamer–antibody sandwich assay. The second one provided lower LOD (1 pg/mL) and better sensitivity.

In summary, the existing aptamers for BNP and NT-proBNP allow the detection of these peptides in biologically and clinically relevant ranges, but lack the well-established ability to perform sandwich assays based fully on aptamers without the involvement of antibodies.

### 2.6. Mid-Regional pro-Adrenomedullin

Adrenomedullin (ADM) is a peptide vasoactive hormone. It is produced by vascular endothelial cells in different tissues; adrenomedullin causes vasodilatation, lowering blood pressure, and maintains vascular integrity [124,125,126]. ADM is increased under HF to decrease the heart overload [125]. Besides HF, hypertension, MI, renal diseases, and sepsis are associated with increased ADM, and the hormone elevation level depends on the severity of vascular damage [126]. The detection of ADM is difficult due to its 22 min half-life time [126]. ADM is synthesized as a preprohormone which is transformed to proADM, and proADM is cleaved to ADM and mid-region-proADM (MR-proADM), which is a 47-amino-acid-long peptide with the molecular weight of 5.1 kDa. MR-proADM can serve as a reliable surrogate biomarker for ADM. MR-proADM is more of a prognostic biomarker than a diagnostic one. MR-proADM can be used for diagnosis and the prognosis of sepsis, septic shock, and organ failure [127]. For the diagnosis of HF, it lacks specificity and does not provide significant diagnostic value compared to NT-proBNP [125]. MR-proADM displays outcome prognostic value in acute severe dyspnea and acute heart failure (ACF) [128] and provides significant prognostic and additive prognostic values to predict outcome and mortality in HF [3]. The normal range of MR-proADM is 0.26–0.51 nM [3]. Cut-off values for MR-proADM are not well established [126]; however, 2.96 ng/mL is reported as the cut-off for prognosis in HF [4,129]. A concentration above 0.72 nmol/L predicts mortality risk in patients with HF [130]. Immunoassay is the basis for MR-proADM detection [131,132], and no aptamers for this biomarker are described in the scientific literature.

### 2.7. Mid-Regional pro-Atrial Natriuretic Peptide

Atrial natriuretic peptide (ANP) is a peptide hormone with diuretic, natriuretic, and hypotensive activity produced by cardiac muscle cells in the walls of the atria in the heart [133,134]. ANP is increased in blood upon tension of the atrial wall, but its half-life is about 2 min [134]. Similar to BNP, ANP is expressed as a part of preprohormone (151 amino acid residues). PreproANP is then processed with the cleavage of the N-terminal fragment to form pro-ANP with a length of 126 amino acids. Finally, pro-ANP is cleaved to form ANP and N-terminal proANR [134]. N-terminal pro-ANP is further enzymatically degraded to several fragments, among which mid-regional proANP (MR-proANP) is formed [135]. MR-proANP contains 38 amino acid residues, has a molecular weight of 4 kDa, and circulates in the blood for about 2 h [3]. Thus, MR-proANP is considered as a stable surrogate biomarker for ANP.

MR-proANP is a diagnostic biomarker and an additive prognostic marker of HF [3,136]. The normal range of MR-proANP is 3.5–61.7 pM [137]. The concentration of MR-proANP lower than 85 pM excludes HF [4,138], and 120 pM is the cut-off for the diagnosis of AHF [139,140]. MR-proANP is recommended as a diagnostic marker for acute HF in the emergency department as an alternative to BNP and NT-proBNP to help differentiate acute HF from non-cardiac causes of acute dyspnea with a cut-off level 0.12 ng/mL [4,141].

Immunoassays are normally used to detect MR-proANP [132,136,142,143]. No aptamers for this peptide are isolated.

### 2.8. Copeptin

Arginine vasopressin (AVP), or the antidiuretic hormone (ADH), plays an important physiological and pathological role. Its main function is the regulation of fluid retention by the kidneys and the maintenance of homeostasis [144]. AVP levels out of normal range are associated with a number of diseases, in particular, HF [145]. However, the detection of AVP is hampered by two factors. First, more than 90% of AVP in the bloodstream is associated with platelets and, therefore, the total number of platelets in the blood and the quality of their precipitation during sample preparation have a significant impact on the accuracy of the analysis. Second, AVP is highly unstable in plasma [144]. AVP is a short peptide consisting of nine amino acids. It is synthesized as part of a preprohormone, which is enzymatically cleaved into AVP, neurophysin II, and copeptin. [146]. Copeptin is a 39-amino-acid glycosylated peptide cleaved from the C-terminus of proAVP. Thus, copeptin is produced in an amount equimolar to AVP. The physiological function of copeptin is unknown. Possibly, it takes part in the folding of vasopressin during the processing of the preprohormone AVP [147]. Copeptin is sufficiently stable in plasma even during long-term storage, which makes it possible to reliably determine it. Thus, copeptin can serve as a surrogate marker for AVP [146]. For healthy individuals and patients with inflammatory diseases, plasma copeptin levels range from 3 to 12 pM [148]. In the case of AMI, its level increases, and ranges from 26 to 160 pM [148]. Copeptin has a prognostic value for the acute stage of AMI, and correlates with the clinical outcome of CHF and AHF [143]. In combination with troponin, the detection of copeptin may be important for the choice of medical strategies [144,145,146,147,148,149] but, on the other hand, copeptin detection along with cTn provides little additive value [3]. Besides, copeptin level is increased with renal failure, and traumatic brain injury and sepsis [3].

Copeptin is detected with immunoassays [150,151,152,153,154,155]. No aptamers for copeptin are isolated, while the L-DNA aptamer for AVP is reported with a K*_D_* value of 1.2 μM [156]. Due to the absence of specific aptamers, copeptin is excluded from aptasensing platforms for biomarker detection.

### 2.9. Interleukin-6

Interleukin-6 (IL-6) is a prominent pro-inflammatory cytokine which regulates cell proliferation, differentiation, and maturation, and plays important role in the immune system and the systemic host defense response to injury [157,158]. IL-6 is a 26 kDa glycosylated protein produced by lymphocytes, neutrophils, eosinophils, B-cells, fibroblasts, mast cells, endotheliocytes, synovial fibroblasts, and macrophages [5,159,160]. Abnormal values of IL-6 are associated with a variety of pathological conditions such as cancer, autoimmune abnormalities, and inflammation [157]. The role of IL-6 in CVD conditions is discussed in special reviews [161,162]. The intracardial expression of IL-6 is activated in ACS and AMI. IL-6 is increased in left ventricular dysfunction (LVD) and CHF alongside with BNP. IL-6 contributes to the development of atherosclerosis [161,162]. IL-6 can serve as a mortality predictor in patients with advanced HF and AMI with cardiogenic shock [163,164]. A normal range of IL-6 is reported to be less than 0.7 pg/mL, rising to 15 ng/mL in HF and increasing even more up to 50 ng/mL with severe inflammation and sepsis [3]. The level of IL-6 in saliva correlates with systemic inflammation [165,166], though local oral inflammation also results in increased salivary IL-6 [167]. Urine levels of IL-6 modestly correlate with its plasma levels, and increased urinary IL-6 is associated with cardio-renal dysfunction indicating tissue-specific inflammation [168].

Several DNA aptamers binding IL-6 are reported. The first study on the isolation of IL-6 aptamer was published in 1995 and revealed the aptamers 5522 and 5523 with K*_D_* in a low micromolar range [169]. The specificity of the aptamers was not addressed, and aptamers 5522 and 5523 were not further applied for the sensing of IL-6, possibly due to poor target binding. A DNA aptamer of 57 nt was reported for the development of an EIS-based aptasensor for IL-6 from serum samples with high selectivity, without referring to the sequence origin [170]. Spiridonova et al. reported another selection of IL-6 aptamers which resulted in the isolation of an aptamer named 12L and its shortened variant 12S [171]. Though this research was not accomplished with non-binding control experiments and specificity studies, the 12L sequence was applied by other researchers for the aptasensor development [172,173]. Based on the 12L aptamer, Tertiş et al. developed an electrochemical aptasensor based on gold and polypyrrole nanoparticles for IL-6 detection in the range of 1 pg/mL–15 mg/mL. The sensor demonstrated good recovery in human serum samples suggesting a sufficient specificity of 12L [172]. Another electrochemical sensor employing a 12L aptamer was capable to detect IL-6 in a range from 1 fM to 100 pM [173]. In this research, non-binding DNA and non-binding protein controls were performed proving the specificity of the 12L aptamer [173]. A bioinformatics research paper describes the docking of experimentally derived DNA sequences to IL-6 [174]. Two potent aptamers, referred to as IL6_2_ and IL6_3_, were recognized, and their binding to IL-6 was qualitatively confirmed by SPR analysis. According to the computer modelling results, these aptamers seemed to bind nearly to the same region of IL-6, thus precluding the development of sandwich assays using them [174]. Later, IL6_2_ was applied for the development of a SERS biosensor detecting mouse IL-6 in a 10 pM–100 nM range with an LOD of 0.8 pM from mouse serum [175]. Several papers describe the development of aptasensors relying on the proprietary aptamer sequences named ATW0077, ATW0082 and ATW0083, offered commercially by Base Pair Biotechnologies, Inc. [175,176,177,178,179]. The sandwich detection of IL-6 is possible using ATW0077/ATW0082 and ATW0077/ATW0083 pairs [176,177]. Base Pair Biotechnologies, Inc. indicate that these aptamers are developed to bind murine IL-6, and the corresponding research papers note the detection of mouse IL-6 as a target [175,178,179] or do not specify the source of the protein [176,177]. The sequence similarity of mouse and human IL-6 is 42% [180]. So far, the applicability of sandwich assays for human IL-6 detection using ATW0077, ATW0082 and ATW0083 aptamers is obscure. Finally, SOMAmers binding IL-6 are reported as its inhibitors [181,182]. No aptamer-based detection of IL-6 from biological fluids other than blood serum has been described.

### 2.10. C-Reactive Protein

C-reactive protein (CRP) is a 125 kDa homopentameric protein produced in liver hepatocytes and some other cell types in response to any inflammation including cardiac damage [3,183,184,185]. CRP is unspecific, but nevertheless it is rather well-established as a cardiac biomarker due to its significant contribution to the pathophysiology of CVD [183]. Elevated CRP levels correlate with the risk of atherothrombotic events [185]. CRP is a strong outcome predictor in AMI, ACS and HF [3]. Normal levels of CRP in the blood serum of healthy individuals are less than 10 ug/mL. Within the normal range, CRP concentration can be used for the risk stratification of CVD. Values less than 1 ug/mL are associated with low CVD risk, levels of 1–3 ug/mL are considered as a medium risk factor, and levels over 3 ug/mL are classified as high risk [186]. During AMI, CRP levels increase above 10 ug/mL. Severe inflammation is associated with CRP levels of about 200 ug/mL.

Laboratory diagnostics of CRP is based on immunological assays [187]. CRP can be detected from saliva, but saliva CRP level does not consistently correlate with its concentration in serum [188]. The detection of CRP in urine is more promising, as it is shown to correlate with serum concentration [189] and has a potential to serve as a predictor for clinical outcomes [189,190].

The number of aptamers targeting CRP is relatively high, possibly due to the widespread use of this biomarker in clinical diagnostics. Two RNA aptamers with K*_D_* values of 125 nM [191] and 2.25 nM have been selected for study [192]. The first one of these RNA aptamers was shown to be specific for CRP using HSA as a cross-target, but the serum assay using SPR biosensor with this aptamer was hampered strongly by IgE [191]. For the second RNA aptamer, no experimental data on its specificity have been provided. Several other DNA aptamers have been described. Aptamer CRP-80-17 characterized with K*_D_* of 3.9 nM was isolated using GO-SELEX by Yang et al. [193]. Its truncated version, named CRP-40-17, with a slightly higher K*_D_* (16.2 nM) but better selectivity is also available [193]. Immobilization via 3′-end was shown to provide a higher response in an SPR-based biosensor for CRP-40-17 [193]. A DNA aptamer named 6th-62-40 was identified using a microfluidic SELEX device [194]. The aptamer exhibited high selectivity to CRP, which was determined against IgG, HSA, Hb, transferrin and myoglobin. Its binding to CRP is characterized by a K*_D_* of 16.2 nM [194]. Another microfluidic aptamer selection experiment by Huang et al. against CRP resulted in a DNA sequence referred to as Clone 1 (K*_D_* value is 3.51 nM) [195]. Its specificity was not examined carefully; an SPR sensor using a Clone 1 aptamer and a CRP-specific antibody was claimed to display high specificity, but no experimental evidence was provided, as the paper was mostly devoted to the development of a microfluidic SELEX chip rather than aptamer validation [195]. Nevertheless, the sensor employing the aptamer Clone 1 was shown to provide good CRP detection in comparison to the established CRP assays from real samples, including whole blood [196]. Another sequence of a CRP-specific aptamer was reported to be derived from the microfluidic SELEX performed by Huang et al. [197,198,199] but, in fact, differed from Clone 1 in the central region and was not presented in the original paper [195]. So far, no K*_D_* has been reported for this aptamer, but several sensors employing it display low LOD, wide linear detection range, good specificity for CRP [197,198,199], and can detect CRP in serum, urine, and saliva [197]. Lai and Hong proposed an interesting protocol employing magnetic fields for aptamer isolation [200,201,202]. These works resulted in the development of several DNA aptamer sequences with K*_D_*s in low nanomolar range, capable of detecting CRP in serum equally to antibodies in ELISA assays [202,203]. Two more DNA aptamers for CRP have been reported for biosensor development without referring to aptamer isolation details. The one produced by OTC Biotech with a length of 20 nt was applied for the SRP-based detection of CRP using an antibody–aptamer sandwich with an LOD of 7 zM (5 fg/mL) [203]. The second one was used for the development of a cell-based biosensor for in vivo CRP detection [204]. No binding characteristics of individual aptamers are available in the literature [203,204]. Finally, the aptamers containing modified nucleobases targeting CRP are described with K*_D_* values of 4 nM (SOMAmer) [205] and 6.2 pM [206]. Thus, at least 17 CRP aptamers are published.

The aptamer-based biosensors for CRP detection have been described and classified in a recent review [207]. The number of papers reporting aptasensors based on a wide range of detection platforms amount to about one hundred. Most of them ensure sufficient LODs, dynamic ranges, and specificity for clinical application in diagnostics. The sensors capable of detecting CRP in whole blood [196,207], saliva [197,206], and urine [197,208] are described. Surprisingly, a single study describes the sandwich assay using two different aptamers [209], and no information on their sequence is provided. So far, aptamer-based sandwich assays for CRP are unavailable, though various antibody–aptamer sandwich assays have been developed [194,195,196,203]. The benefits of sandwich-type assays are well illustrated by Wu et al. [194]. The SPR sensor using the 6th-62-40 aptamer was developed in three variants assuming the direct detection of CRP by the aptamer, the sandwich detection of CRP by the aptamer, the CRP-specific antibody, and the sandwich detection of the aptamer and the CRP-specific antibody with signal amplification, providing LODs of 1, 0.1 nM, and 10 pM, respectively. Moreover, the best zeptomolar LOD of CPR aptasensors was also obtained using sandwich detection [203].

The DNA aptamers CRP-40-17 [193], 6th-62-40 [194], Clone 1 [195], and an oligonucleotide referenced in [197,198,199] exhibit the highest potential for the clinical detection of CRP in the future, because of their good K*_D_* values and specificity. The search for aptamer sandwich combinations targeting CRP is required.

### 2.11. Tumor Necrosis Factor Alpha

Tumor necrosis factor alpha (TNFα) is a cytokine which acts as an intercellular chemical messenger in inflammatory processes. It is produced by a variety of cells, mostly immune, but also neural, skin, and endothelial, in a form of transmembrane TNFα, which is then cleaved by a specific enzyme to release soluble TNFα [210]. Both transmembrane and soluble forms of TNFα are biologically active as homotrimers, with a molecular weight of 51 kDa, and each subunit being of 17 kDa [211]. Playing a primary role in immune regulation, it poses an influence on many other physiological pathways [5] including vascular function [212]. Increases in TNFα level are associated with vascular inflammation, oxidative stress, atherosclerosis, thrombosis, vascular remodeling, and endothelium apoptosis [212]. TNFα cannot serve for diagnostics of CVDs because its level is increased in almost any inflammatory condition, but it can be used as a mortality predictor in HF [3,163]. TNFα level depends on age and sex [163]: a normal range is reported to be 0.7 ± 0.3 pg/mL [213]. Levels of TNFα in the range of 1–10 pg/mL is detected in MI and HF [3]. TNFα can be detected in saliva, but salivary level and its correlation to blood concentration in CVD have not yet been studied [214]. TNFα concentration in urea was shown not to differ significantly in patients with severe CHF, making the detection of TNFα in urea a possible practical instrument for the non-invasive diagnosis of CVD [215].

Several DNA aptamers and one RNA aptamer targeting TNFα are known, and most of them were introduced as TNFα inhibitors for anti-inflammatory therapy [216,217,218,219,220]. The VR11 aptamer is one of the most popular DNA ligands, and its binding to TNFα is characterized by the K*_D_* value of 7 nM [216]. VR11 inspired the development of biosensors for electrochemical [221,222], FET-based [223], and the optical [224,225] detection of TNFα. The conjugation of the VR11 aptamer with the TNFα-specific antibody is often used to design sandwich detection schemes [221,225]. An RNA aptamer named T3.11.7 [220] has also been applied for the electrochemical detection of TNFα [226,227]. Two more DNA aptamers with a length of 41 [218] and 49 nt (T1-4) [219] have not been applied for sensing yet, but have proved TNFα binding and are potent TNFα inhibitors. The T1-4 ligand is, in fact, a dimer of two shorter aptamers named T1 and T4, which are assumed to bind different sites in the TNFα structure [219], and thus show promising potential for dual-aptamer sandwich application. An isolation of the aptamer S01 binding to TNFα with K*_D_* of 0.19 nM is described in [228]. S01 is reported to be superior to the VR11 aptamer and the TNFα-specific antibody mAb11 in terms of affinity and the efficiency of target detection in serum using the ELISA protocol [228]. Surprisingly, we did not find the aptasensors for TNFα employing this aptamer. Finally, an electrochemical biosensor for TNFα with good analytical characteristics is described which engages a unique DNA aptameric sequence [229]. The origin of this aptamer is not specified or referred to.

In summary, the range of established aptamers for TNFα is wide enough, but these aptamers have resulted in few aptasensors. The developed sensors mostly exhibit LOD and linear ranges suitable for real TNFα measurement. Aptasensors are reported to detect TNFα in whole blood [226,227] and serum [221,228]. No dual-aptamer sandwich assays have been reported and no detection of TNFα from the urine or saliva using these aptamers has been described. VR11 [216] and S01 [228] DNA aptamers for TNFα are the most perspective for analytical application. VR11 is well established for TNFα sensing, and S01 exhibits the best K*_D_* value. T1 and T4 aptamers are of specific interest for the development of sandwich assays.

### 2.12. Galectin-3

Galectin-3 is a carbohydrate binding protein that plays many important regulatory roles in inflammation, immunity, and cancer. Recent studies demonstrates that Galectin-3 overexpression and secretion is associated with several diseases, and it is extensively studied in the context of fibrosis, HF, atherosclerosis, and diabetes mellitus [230]. Regarding HF, the increase in concentration is associated with an increased risk of mortality. The first data about Gal-3 circulating levels and HF were generated using ELISA [231]. Today, Gal-3 measurements are performed by commercial automated immunoassays [232]. The Gal-3 concentrations range, in healthy individuals, from 6.9 to 20.8 ng/mL [233]. Independent of symptoms and other laboratory investigations, levels of Gal-3 higher than 25.9 ng/mL predict a patient who is likely to have a rapid progression of HF with a very high risk of hospitalization and death [234]. However, successful treatment is associated with decreasing Gal-3 levels [235], indicating that Gal-3 control has a potential to be integrated into the management of patients with HF. Salivary levels of Gal-3 moderately correlate with serum levels and are significantly increased in patients with HF. The risk of cardiovascular death or hospitalization is strongly increased if the salivary level of Gal-3 is higher than 172.58 ng/mL [214].

To date, no aptamers with affinity to Gal-3 have been found, and the emergence of new aptamer-based methods is a matter of the near future. Moreover, recent studies indicate that Gal-3 is involved in cardiovascular fibrosis as a regulatory molecule in HF [230,236] and, thus, the development of aptamers for Gal-3 inhibition may be a promising direction in findings new drugs.

### 2.13. Soluble Suppression of Tumorigenicity 2

The suppression of tumorigenicity 2 (ST2) belongs to the IL-1 receptor family and exists as two important isoforms: transmembrane (ST2L) and soluble (sST2), which are produced by alternative splicing [237,238]. The transmembrane form binds to IL-33, the cytokine that is secreted by cells in response to tissue damage and has a cardioprotective effect. The soluble form acts as a decoy receptor, limiting the pro-inflammatory action of IL-33 [239]. When circulating sST2 levels are elevated, the cardioprotective action of IL-33 is reduced [240]. Hence, sST2 is a biomarker of vascular health with diagnostic and/or prognostic value in various CVDs, including coronary artery disease, MI and atherosclerosis [241]. sST2 was found to be a strong, independent predictive factor for HF and also to improve risk stratification accuracy for HF events in combination with NT-proBNP and troponin T [240,242]. A. Aleksova et al. proposed a practical tool based on sST2-assisted flowcharts in order to aid emergency medicine physicians to determine three common clinical scenarios [243]. Though IL-33 can be detected in the blood, saliva and urine, only serum values of sST2 can be derived from the scientific literature. The cut-off value of 35 ng/mL is the most frequently reported for HF, whereas the study discloses that the risk of all-cause death, cardiovascular death, and HF hospitalization increases exponentially with sST2 levels higher than 28 ng/mL [244,245,246]. sST2 level depends on sex and lower cut-offs for risk stratification could be used for women [247]. In order to aid the prognosis of HF at the point of care, a duplex platform targeting BNP and sST2 was developed [4,248]. Still, the most common method for sST2 measurement is the ELISA assay, although it is not clear which epitopes are detected by the antibodies against sST2 [249].

### 2.14. Growth Differentiation Factor 8 (Myostatin)

Myostatin (growth differentiation factor 8, GDF8), a cytokine from the transforming growth factor-β (TGF-β) family, inhibits skeletal muscle growth [250,251]. GDF8 active protein is a 25 kDa homodimer expressed in skeletal muscle and in heart and adipose tissue [250,252]. Myostatin expression in cardiomyocytes is increased in patients with HF [252] and AMI [253], and myostatin concentration in plasma positively correlates with cTnI [254] and natriuretic peptide [255] levels. Besides, the elevation level of myostatin reflects the extent of cardiac damage and correlates with adverse disease outcomes [254,255,256]. GDF8 level in healthy people is 10–80 ng/mL, and in CHF this is increased to 30–105 ng/mL [3]. On the other hand, myostatin serum levels in non-cardiac ICU patients are shown to negatively correlate with markers of systemic inflammation, including CRP and IL-6 [257] and, in this case, lower GDF8 levels of about 10 ng/mL are a predictor of unfavorable prognosis [3,257]. The detection of GDF8 from saliva or urine is not reported.

Common myostatin measurement is based on immunoassays [255,256,257,258]. The high structural similarity of GDF8 and GDF11, proteins of the TGF-β family, encumbers the accurate detection of myostatin [259]. The SOMAmer binding both GDF8 and GDF11 has been reported [260]. Recently, it was established that SOMAmers could selectively bind to each of these proteins [261]. No other aptamers except SOMAmers are known to bind myostatin.

### 2.15. Growth Differentiation Factor 15

Growth differentiation factor 15 (GDF15) is a stress-responsive cytokine which belongs to the TGFβ family. It is a 25 kDa homodimer in which the monomers are linked by a disulfide bond [262]. GDF15 is abundantly expressed in the liver, intestine, kidneys, and placenta [263]. In the heart, the expression of GDF15 is normally low but increases in response to cardiovascular injury induced by pressure overload, HF, ischemia–reperfusion injury, and atherosclerosis [263,264,265]. Increases in GDF15 are also associated with a large variety of diseases including inflammation, cancer, and obesity [265]. GDF15 seems to exhibit protective and anti-inflammatory effects in pathological processes including CVD, though the detailed biochemical mechanism of its functioning is still under investigation [262,263]. GDF15 is proposed to serve as a biomarker of disease progression [263,264,265]. GDF15 is associated with unfavorable prognosis in CVD [263]. Normal GBF15 level is 0.1–1.2 ng/mL. Levels of 1.2–1.8 ng/mL are considered moderately elevated, levels higher than 1.8 ng/mL^−1^ are considered severely elevated, reaching 47 ng/mL in patients with severe diseases [3].

GDF15 detected from saliva could not discriminate between patients with AMI and healthy controls, and no correlation between salivary and serum GDF15 was detected [266]. The studies of urinary GDF15 levels demonstrate its association with blood concentration, but were conducted for the patients with renal dysfunction [267,268,269,270].

The SOMAmer-based assay has been reported for GDF15 detection [271], although no other aptamers have been identified.

**Table 1:** Aptamers for cardiac biomarkers.

Biomarker	Aptamer Name, Type, Length	Aptamer Sequence, 5′-3′ Direction	K*_D_*	Method for Affinity Estimation	Reference
cTnI	Tro4, DNA, 40 nt	CGTGCAGTACGCCAACCTTTCTCATGCGCTGCCCCTCTTA	270 pM	SPR	[20]
	Tro6, DNA, 40 nt	CGCATGCCAAACGTTGCCTCATAGTTCCCTCCCCGTGTCC	317 pM	SPR	[20]
	TnIApt23, DNA	No sequence published	2.7 nM	Fluorescence	[36]
	TnIApt19, DNA	No sequence published	6.3 nM	Fluorescence	[36]
	TnIApt18, DNA, 79 nt	GCCTGTTGTGAGCCTCCTAACTACATGTTCTCAGGGTTGAGGCTGGATGGCGATGGTGGCATGCTTATTCTTGTCTCCC	9 nM	Fluorescence	[36]
	TnIApt11, DNA, 79 nt	GCCTGTTGTGAGCCTCCTAACTTCAAGGTGTGGTCAGTCTTGGATTGGAGGAGTATGNGCATGCTTATTCTTGTCTCCC	10.25 nM	Fluorescence	[36]
	TnI2, DNA, 72 nt	GGCAGGAAGACAAACACCCAACCGAGGATGCAACGCTTGTTGTCATACTGTGATGTTGGTCTGTGGTGCTGT	19.8 nM	SPR	[39,40]
	Apt 3, DNA, 96 nt	CGTACGGTCGACGCTAGCCGGACACCCAAGTCAGACGTGCCCATTATCGCGCGATACGTATTATTTCTTGCTCGGGGCCACGTGGAGCTCGGATCC	1.01 nM	ELONA	[41]
	Apt 6, DNA, 96 nt	CGTACGGTCGACGCTAGCCCGGAGCGAAGGCGGCCCCGTTTGCGTGCAGCGTAGTCTGTAGACAACAGTGCTGTGGGCCACGTGGAGCTCGGATCC	0.68 nM	ELONA	[41]
	A4, L-DNA, 76 nt	AGTCTCCGCTGTCCTCCCAGTGCAGGCTGAGTGGGTGGGTGGGTGGTGTGGCCACGTTGGGATGACGCCGTGACTG	3.5 nM	SPR	[18]
	B10, L-DNA, 76 nt	AGTCTCCGCTGTCCTCCCGATGCACTTGACGTATGTCTCACTTTCTTTTCATTGACATGGGATGACGCCGTGACTG	10.7 nM	SPR	[18]
	A6, L-DNA, 76 nt	CAGTGAGTGATGGTGAGGGCTTAGTTCGCCGCTCATGCCGAATCTCCTGTATAAATACCCACACTGTCCATACACG	540 pM	SPR	[42]
	C6, L-DNA, 76 nt	CAGTGAGTGATGGTGAGGGTGAATCGGTGTCGACTATTAAATTAAGTTGTGGTTGTTCCCACACTGTCCATACACG	305 pM	SPR	[42]
	DNA, 28 nt	CCAATGCAGTGGGGAGGGACTGCGTTGG	nd *	nd	[47]
	biotin-apta, DNA, 80 nt	TTCAGCACTCCACGCATAGCTCAGCCGGCAATGAACAACCTCCATTCTAACGCAGTGTTACCTATGCGTGCTACCGTGAA	nd	nd	[48]
	TnAp1, DNA, 80 nt	GGCAGCAGGAAGACAAGACATGGGTGGCGGGGACGGGGCGATGGGAACTTAGATTGCTAGTGGTTCTGTGGTTGCTCTGT	61.51 nM	Bioluminescence	[46]
	TnAp2, DNA, 80 nt	GGCAGCAGGAAGACAAGACAGGCAGTGTCACGCGCTCAAGGGTGGAGGGGTCGGGGAGGTTGGTTCTGTGGTTGCTCTGT	42.01 nM	Bioluminescence	[46]
	TnAp4, DNA, 80 nt	GGCAGCAGGAAGACAAGACACAACGCATGGGTGGGACGACGGGTGGGCAAGAGACACGCCTGGTTCTGTGGTTGCTCTGT	167.1 nM	Bioluminescence	[46]
	TnAp5, DNA, 80 nt	GGCAGCAGGAAGACAAGACACACGGGAGGGAGGGTAGGGTGTGTGTCGAATCACTGCGCATGGTTCTGTGGTTGCTCTGT	255.7 nM	Bioluminescence	[46]
	TnAp10, DNA, 80 nt	GGCAGCAGGAAGACAAGACACCACATCTATGGGTGGGACGATGGGTGGGCCGAAACGACCTGGTTCTGTGGTTGCTCTGT	121.4 nM	Bioluminescence	[46]
	TnAp12, DNA, 80 nt	GGCAGCAGGAAGACAAGACATCGGGAGGGAGGGAGGGCAGTCTAGTCTCATGTGTTTCCATGGTTCTGTGGTTGCTCTGT	24.16 nM	Bioluminescence	[46]
	TnAp14, DNA, 80 nt	GGCAGCAGGAAGACAAGACACTACCCATACACTTAGGGACGGGTGGCCGGGGAGGGAGGTTGGTTCTGTGGTTGCTCTGT	79.04 nM	Bioluminescence	[46]
	TnAp2t1, DNA, 40 nt	GGCAGTGTCACGCGCTCAAGGGTGGAGGGGTCGGGGAGGT	39.06 nM	Bioluminescence	[46]
	TnAp2t2, DNA, 54 nt	AGACAAGACAGGCAGTGTCACGCGCTCAAGGGTGGAGGGGTCGGGGAGGTTGGT	24.93 nM	Bioluminescence	[46]
	TnAp2t3, DNA, 27 nt	GCTCAAGGGTGGAGGGGTCGGGGAGGT	30.6 nM	Bioluminescence	[46]
	DNA, 55 nt	No sequence published	nd	nd	[49]
	DNA	No sequence published	nd	nd	[50]
cTnT	Tro 4, DNA, 40 nt	CGTGCAGTACGCCAACCTTTCTCATGCGCTGCCCCTCTTA	nd	nd	[57,58]
	Apt.1, DNA, 71 nt	ATACGGGAGCCAACACCAGGACTAACATTATAAGAATTGCGAATAATCATTGGAGAGCAGGTGTGACGGAT	122 nM	SPR	[59]
	Apt.2, DNA, 71 nt	ATCCGTCACACCTGCTCTCCAATGATTATTCGCAATTCTTATAATGTTAGTCCTGGTGTTGGCTCCCGTAT	190 nM	SPR	[59]
	AraHH001, DNA, 40 nt	ACGTACCGACTTCGTATGCCAACAGCCCTTTATCCACCTC	43 nM	Flowcyto- metry	[61,62]
Mb	Myo40-7-27, DNA, 40 nt	CCCTCCTTTCCTTCGACGTAGATCTGCTGCGTTGTTCCGA	4.93 nM	SPR	[75]
	Myo40-7-69, DNA, 40 nt	CGAGTACTTCTTTGCTAGTTCGCGAGATACGTTGGCTAGG	6.38 nM	SPR	[75]
	Myo40-7-34, DNA, 40 nt	ACGCACAATTCCTTGTCCAATTAGGAAATTCTACGCGGAT	5.58 nM	SPR	[75]
	Mb 089, DNA, 72 nt	ATCCGTCACACCTGCTCTTAATTACAGGCAGTTCCACTTAGACAGACACACGAATGGTGTTGGCTCCCGTAT	nd	nd	[80]
	ST1, DNA	No sequence published	65 pM	SPR	[81]
	DNA, 78 nt	ATCCAGAGTGACGCAGCACAACGTGCAAATTATACCTGTTTTCCCCTTTTCCTACAAGTGCTATGGACACGGCTTAGT	65 pM	nd	[83]
CK-MB	C.Apt.21, DNA, 45 nt	GGGGGGTGGGTGGGGGATCTCGGAGGATGCTTTTAGGGGGTTGGG	0.81 nM	ELONA	[96]
	C.Apt.30, DNA, 43 nt	CATTGAGAGGGGGTGGCCGTAGTCAGGTGGGTGGGGGTTTGAG	24.04 nM	ELONA	[96]
hFABP	N13, DNA, 90 nt	CACCTAATACGACTCACTATAGCGGATCCGAAGGGGGCGCGAGGTGTAAGGGTGTGGGGTGGTGGGTGGGCCTGGCTCGAACAAGCTTGC	74.3 nM	CD	[101]
	N53, DNA, 90 nt	CACCTAATACGACTCACTATAGCGGATCCGAGGGGGTAGCGGGTGGGCCGGTG_GATGCGGGGCGCCGGCGCCTGGCTCGAACAAGCTTGC	333.7 nM	CD	[101]
BNP	A10, DNA, 40 nt	GGCGATTCGTGATCTCTGCTCTCGGTTTCGCGTTCGTTCG	12 nM	Fluorescence	[121]
	A8, DNA, 40 nt	CGAAATACACAGCCAGGACTGGAGGGCAAGGGTAACGAGC	139.4 nM	Fluorescence	[121]
	A11, DNA, 40 nt	TGAGCCCGGGACAGAGAGACCGGACCACGTGCCCGGGCC	28 nM	Fluorescence	[121]
	A14-1, DNA, 40 nt	ATAACGACATCCGCCGGCACGAAGGGATCAAGTCGATAGG	22.4 nM	Fluorescence	[121]
	A14-5, DNA, 40 nt	CCCGTGCTTTGGCCCTCCATGCAGCCTTGAGCCTATGCC	104.6 nM	Fluorescence	[121]
	8–12, DNA, 50 nt	TAAACGCTCAAAGGACAGAGGGTGCGTAGGAAGGGTATTCGACAGGAGGCTCACA	nd	ELONA	[115]
	2F, DNA, 72 nt	ATACGGGAGCCAACACCATGGTGGGTACTACCCTTAAAAACATCGCCCCCTACGAGAGCAGGTGTGACGGAT	nd	ELONA	[120]
	6R, DNA, 72 nt	ATCCGTCACACCTGCTCTCGTAGGGGGCGATGTTTTTAAGGGTAGTACCCACCATGGTGTTGGCTCCCGTAT	nd	ELONA	[120]
	14bF, DNA, 72 nt	ATACGGGAGCCAACACCACCTATTACAGACCCAATTTCCACCTGGCATTTCTATAGAGCAGGTGTGACGGAT	nd	ELONA	[120]
	25cF, DNA, 72 nt	ATACGGGAGCCAACACCACCTCTCACATTATATTGTGAATACTTCGTGCTGTTTAGAGCAGGTGTGACGGAT	nd	ELONA	[120]
NT-proBNP	N20a, DNA, 72 nt	GGCAGGAAGACAAACAGGTCGTAGTGGAAACTGTCCACCGTAGACCGGTTATCTAGTGGTCTGTGGTGCTGT	2.89 nM	SPR	[39,123]
IL-6	5522, DNA, 59 nt	CTCATAAGTCGTTGCAACCCCGTGCGCATGGACTGATCTTCCGCLGAATCACGAGGGTA	6.15 µM	GMSA	[169]
	5523, DNA, 56 nt	CCTCACGAACCATGATCACGCACCAACCAGGCCGTGTTAAAGAGGGCACACTGTAT	1.25 µM	GMSA	[169]
	DNA, 57 nt	GTCTCTGTGTGCGCCAGAGACACTGGGGCAGATATGGGCCAGCACAGAATGAGGCCC	nd	nd	[170]
	12L, DNA, 31 nt	GGTGGCAGGAGGACTATTTATTTGCTTTTCT	17 nM	SPR	[171]
	12S, DNA, 16 nt	GGTGGCAGGAGGACTA	190 nM	SPR	[171]
	IL62, DNA, 30 nt	CTTCCAACGCTCGTATTGTCAGTCTTTAGT	nd	nd	[174]
	IL63, DNA, 30 nt	CTTCCGTGAAACCAACGTGCCCTCAATCCG	nd	nd	[174]
	ATW0077, DNA, 32 nt	No sequence published	5.4 nM	nd	[178,179]
	ATW0082	No sequence published	nd	nd	[176]
	ATW0083	No sequence published	nd	nd	[177]
CRP	DNA, 20 nt	GGGCCTCCGGTTCATGCCGC	nd	nd	[203]
	DNA, 72 nt	GGCAGGAAGACAAACACACAAGCGGGTGGGTGTGTACTATTGCAGTATCTATTCTGTGGTCTGTGGTGCTGT	nd	nd	[204]
	CRP-80-17, DNA, 80 nt	AGCAGCACAGAGGTCAGATG CCCCCGCGGGTCGGCTTGCCGTTCCGTTCGGCGCTTCCCC CCTATGCGTGCTACCGTGAA	3.9 nM	SPR	[193]
	CRP-40-17, DNA, 40 nt	CCCCCGCGGGTCGGCTTGCCGTTCCGTTCGGCGCTTCCCC	16.2 nM	SPR	[193]
	6th-62-40 DNA, 40 nt	CGAAGGGGATTCGAGGGGTGATTGCGTGCTCCATTTGGTG	16.2 nM	SPR	[194]
	Clone 1 DNA, 72 nt	GGCAGGAAGACAAACACGATGGGGGGGTATGATTTGATGTGGTTGTTGCATGATCGTGGTCTGTGGTGCTGT	3.51 nM	SPR	[195]
	DNA, 72 nt	GGCAGGAAGACAAACATATAATTGAGATCGTTTGATGACTTTGTAAGAGTGTGGAATGGTCTGTGGTGCTGT	nd	nd	[197,198,199]
	CRP1-1 RNA, 104 nt	GGGCGAAUUCGGGACUUCGAUCCGUAGUACCCACCAGGCAUACACCAGCACGCGGAGCCAAGGAAAAAUAGUAAACUAGCACUCAGUGCUCGUAUGCGGAAGCU	2.25 nM	SPR	[192]
	RNA, 44 nt	GCCUGUAAGGUGGUCGGUGUGGCGAGUGUGUUAGGAGAGAUUGC	125 nM	nd	[191]
	F27K-4 DNA, 60 nt	AGCAGCACAGAGGTCAGATGGCCCCCGAAGTTGCTTAGTCCCTATGCGTGCTACCGTGAA	22.71 nM	qPCR	[200]
	>27K-1 DNA, 60 nt	AGCAGCACAGAGGTCAGATGTCTGTAATTTATAGTTCCATCCTATGCGTGCTACCGTGAA	7.65 nM	qPCR	[200]
	20N AC > 200 K DNA, 60 nt	AGCAGCACAGAGGTCAGATGAATTACAAATTTGGACTGTTCCTATGCGTGCTACCGTGAA	8.35 nM	qPCR	[201]
	20N AC F200K-1 DNA, 60 nt	AGCAGCACAGAGGTCAGATGGCATTGTATCACAGGTACTGCCTATGCGTGCTACCGTGAA	12.49 nM	qPCR	[201]
	sOS-AC-20N-1 DNA, 60 nt	AGCAGCACAGAGGTCAGATGGATACCAAGGTCCGCTGGTTCCTATGCGTGCTACCGTGAA	5.96 nM	qPCR	[201]
	OS-AC-20N-3 DNA, 60 nt	AGCAGCACAGAGGTCAGATGCGCTTGTGATGGGTGATGGGCCTATGCGTGCTACCGTGAA	5.70 nM	qPCR	[201]
	PF20N-RO-MARAS-84-1 DNA, 20 nt	GTTGACGGGCGATTGGTCTT	23.58 nM	qPCR	[202]
TNFα	DNA, 41 nt	GCGCCACTACAGGGGAGCTGCCATTCGAATAGGTGGGCCGC	8 nM	qPCR	[218]
	VR11, DNA, 25 nt	TGGTGGATGGCGCAGTCGGCGACAA	7 nM	SPR	[216]
	T3.11.7, 2′-NH2-RNA, 28 nt	GGAGUAUCUGAUGACAAUUCGGAGCUCC	nd	ELONA	[220]
	T1-4, DNA, 49 nt	TCCGATCGGTATATCCGTCGGATTTTTTTTTTGGTCACTGCATGTGACC	67 nM	Cell cytotoxicity assay	[219]
	T1, DNA, 17 nt	GGTCACTGCATGTGACC	195 nM	Cell cytotoxicity assay	[219]
	T4, DNA, 22 nt	TCCGATCGGTATATCCGTCGGA	142 nM	Cell cytotoxicity assay	[219]
	S01, DNA, 81 nt	ATCCAGAGTGACGCAGCATGCTTAAGGGGGGGGCGGGTTAAGGGAGTGGGGAGGGAGCTGGTGTGGACACGGTGGCTTAGT	0.19 nM in buffer 0.27 nM in serum	FACS	[228]
	B01, DNA, 80 nt	ATCCAGAGTGACGCAGCAGGTTAAGGTGTAGGTCCGGGTGGGGGGGTGGGTTGGGGGACTGGTGGACACGGTGGCTTAGT	0.35 nM	FACS	[228]
	DNA, 33 nt	GCGGCCGATAAGGTCTTTCCAAGCGAACGAAAA	nd	nd	[229]

* no data.

## 3. SOMAmers Targeting Cardiac Biomarkers

Being systematically developed by L. Gold’s group, SOMAmers were developed for a few thousand protein targets [272,273], with almost all the above-discussed cardiac biomarkers being among them [274], with the possible exception of neurohumoral markers (copeptin, MR-proADM, MR-proANP) as we did not find any mention of these in the scientific literature. SOMAscan is a commercial solution in which SOMAmers are used to analyze their targets in a sample [274]. SOMAscan is now applied as a high-throughput proteomics platform for the recognition of biomarkers based on the qualitative comparison of disease and control cohorts’ inputs, but it has potential for the quantitative measurements of biomarkers [274,275]. We did not include SOMAmer sequences, which are published for some biomarkers such CRP or IL-6, in this review, mainly due to the complexity of their synthesis. Regardless, the existence and success of SOMAscan solutions encourage the future real implementation of aptamers to routine diagnostics and medicinal research.

## 4. Future Research Directions

Aptamers have a real potential to displace antibodies in routine biochemical assays in the future but, today, aptamer-based detection is rather far from real practical implementation, at least regarding cardiovascular biomarkers. Table 2 illustrates the current state of aptamer research in respect to the detection of CVD biomarkers discussed in this review. DNA aptamers for cardiac biomarkers are prevalent over RNA ones. The primary reason for this seems to be due to the less laborious protocol for DNA aptamer isolation compared to RNA. However, DNA is more convenient for analytic purposes because of its higher thermal and nuclease stability, accompanied by a lower synthesis cost.

For cTnI, cTnT, myoglobin, CK-MB, BNP, NT-proBNP, IL-6, CRP, and TNFα, the aptamers are known which enable the detection of these biomarkers in clinically relevant ranges with good specificity. For HFABP, the existing aptamers do not allow detection in clinical ranges. No DNA/RNA aptamers are known for MR-proADM, MR-proANP, Copeptin, sST2, Gal3, GDF8, or GDF15. This restricts the transition from antibodies to aptamer-sensing platforms.

Dual-aptamer-based sandwich detection is available for cTnI (Tro 4 + Tro 6 and Apt 3 + Apt 6 are the best choices), cTnT, and CK-MB. For myoglobin, a split aptamer sensing strategy has been developed. This assay cannot serve for detection schemes assuming separated binding and detection steps such as ELONA, because a single part of a split aptamer cannot bind the target. The dual-aptamer sandwich assay for HFABP needs further development to improve its dynamic range. The sandwich assay for BNP lacks selectivity. For IL-6, both known aptamer pairs were initially developed for murine protein, and its application for human IL-6 is not obvious. The sequences of the aptamers used for CRP sandwich detection have not been provided. So far, dual-aptamer-based sandwich assays are unavailable for most cardiac biomarkers, while sandwich-type assays offer a strong basis for the universalization of single-analyte detection and for simultaneous multiple-target detection.

The detection of cardiac biomarkers from saliva, urine, and whole blood, in addition to serum, is demanded for POC testing. As derived from the literature, sensing from the saliva, in respect to CVDs, makes sense for troponins, myoglobin, CK-MB, BNP, NT-proBNP, and Gal-3, while detection from urine is appropriate for CRP. Among these biomarkers, troponins and BNP/NT-proBNP are of special interest for POC testing because these are the most popular biomarkers which can serve for diagnostics in acute settings. Aptamer-based detection from saliva is demonstrated only for troponins and myoglobin. The aptasensing of CRP from urine has also been described. The detection of BNP and NT-pro-BNP from saliva needs to be accomplished in the future. The practical implementation of aptamer-based POC devices appears to promise success in the near future; however, in this field, more efforts are required to gain knowledge of non-invasive CVD biomarker detection. This poses the need for both aptamer and medicinal research.

Aptamer-based assays for some popular cardiac biomarkers, such as cTnI, are offered within a huge variety of detection platforms, but the practical implementation of aptamer assays to diagnostics relies, firstly, on the unification of the detection platform and analysis schemes for the wider range of biomarkers, rather than on their diversification, as this can be learned from SOMAscan products and the commercial immunoassays normally used in laboratory detection.

So far, the following research directions can be derived to fulfill the gaps in the aptamer-based sensing of cardiac biomarkers:

- The development of novel aptamers binding to MR-proADM, MR-proANP, Copeptin, sST2, Gal3, GDF8, and GDF15. During aptamer selection, specificity should be addressed using correct negative and counter targets, which should include proteins with similar structures, other biomarkers known to increase in the same disease conditions, and the most abundant proteins in the medium desired for detection, e.g., albumins and globulins for the detection from serum. Selection conditions should maximally imitate the intended aptamer usage;
- The characterization of novel—and some already existing—aptamers. This includes the confirmation of binding, for which using several different methods is desired, the evaluation of cross-specificity, and the study of the influence of the experimental conditions on the aptamer–target binding (pH, buffer composition, temperature);
- The development of sandwich detection assays based on aptamers. For this purpose, the existing aptamers can be systematically paired for the evaluation, and novel aptamers can be isolated using specific selection procedures intended to develop the aptamers recognizing different binding sites of the same target;
- The characterization and validation of aptamer-based assays. LOD and dynamic detection ranges of sensors must allow the detection of biomarkers in clinically relevant ranges. The detection of the targets from real samples is strongly recommended, and the results should be compared with the validated alternative assays. The performance of the aptamer-targeting cardiac biomarkers from different biological fluids (saliva, urine, whole blood) is of specific interest for the development of POC biosensors. The range of analytical targets for the same analysis scheme should be maximally extended, because the universal assay platforms allowing the detection of multiple biomarkers within single or parallel measurements are required both for laboratory instrumental diagnostics and POC devices;
- The implementation of the developed aptamer-based assays to clinical studies, alongside the validated assays. The assays should be carefully characterized with specificity, sensitivity, and accuracy. The cut-off levels for each assay should be examined individually, as their values may differ for different assays in some cases. Moreover, the combination of multiple biomarkers is an increasing trend in diagnostics and prognostics, and especially for the risk stratification of CVDs. Aptamer-based multiple-biomarker detection should be implemented more intensively to respective clinical studies;
- Further medicinal research of salivary CVD biomarkers. The detection from saliva is a very attractive non-invasive method of CVD diagnostics using aptamers, but some pitfalls associated with saliva collection, storage, and analysis exist, which impede the routine use of saliva in clinical practice [19,214,276]. Aptamer-based assays specifically developed to detect cardiac biomarkers from saliva can assure the required sensitivity to measure lower biomarker concentrations in saliva compared to serum, and can thus assist to establish cut-off levels;
- The further development and promotion of aptamer-based POC devices. Such devices should have low cost, convenient and affordable signal read-outs (such as visual or smartphone-assisted detection), and easy result interpretation for the unexperienced end-user. Both diagnostics and risk assessment devices are in demand.

We believe that overcoming these experimental issues will result in the real practical implementation of aptamers to routine clinical diagnostics.

## 5. Conclusions

A lot of effort in the field of aptamer research has resulted in the development of aptamer-based detection for cardiac biomarkers. This research is non-uniformly distributed among the known CVD biomarkers, with excessive respect to troponins, and lacking attention given to some other biomarkers such as neurohormones. The selection of novel aptamers and the development of aptasensors employing a universal detection scheme for the analysis of a wider range of biomarkers are required to assist the future expansion of aptamers and aptamer-based sensing to practical usage in clinical diagnostics and medicinal research.

## Figures and Tables

**Table 2:** Summary of aptamer-based detection methods for the most common cardiac biomarkers.

Biomarker	Aptamer	Aptasensing in Relevant Concentration Range	Dual-Aptamer-Based Sandwich Detection	Aptasensing in Biological Fluids			
				Serum	Whole Blood	Saliva	Urine
cTnI	DNA, L-DNA	+	Tro4 + Tro6 Apt 3 + Apt 6 B10 + C6 (L-DNA) TnAp2t3 + TnAp10	+	+	+	+
cTnT	DNA	+	Apt.1 + Apt.2	+	−	+	+
Myoglobin	DNA	+	Split aptamer Myo40-7-27	+	−	+	+
CK-MB	DNA	+	C.Apt.21 + C.Apt.30	+	−	−	−
HFABP	DNA	−	N13 + N53	−	−	−	−
BNP	DNA	+	25c + 2F, poor selectivity	+	+	−	−
NT-proBNP	DNA	+	−	+	−	−	−
MR-proADM	−	−	−	−	−	−	−
MR-proANP	−	−	−	−	−	−	−
Copeptin	−	−	−	−	−	−	−
IL-6	DNA	+	IL6_2_ + IL6_3_ ATW0077 + ATW0082/ATW0082, mouse IL-6-specific	+	−	−	−
CRP	DNA, RNA	+	+ No aptamer sequences available	+	+	−	+
TNFα	DNA, RNA	+	−	+	+	−	−
Gal-3	−	−	−	−	−	−	−
sST2	−	−	−	−	−	−	−
GDF8	−	−	−	−	−	−	−
GDF15	−	−	−	−	−	−	−

cTnI—cardiac troponin I; cTnT—cardiac troponin T; CK-MB—creatine kinase; BNP—B-type natriuretic peptide; NT-proBNP—N-terminal pro-B-type natriuretic peptide; MR-proADM—mid-regional proadrenomedullin; MR-proANP—mid-regional pro-atrial natriuretic peptide; IL-6—interleukin-6; CRP—C-reactive protein; TNFα—tumor necrosis factor alpha; sST2—soluble suppression of tumorigenicity 2; Gal3—galectin-3; GDF8—growth differentiation factor 8; GDF15—growth differentiation factor 15. “−”—not known, “+”—known.

## Abbreviations

ACS	acute coronary syndrome
ADH	antidiuretic hormone
ADM	adrenomedullin
AMI	acute myocardial infarction
ANP	atrial natriuretic peptide
AVP	arginine vasopressin
BNP	B-type natriuretic peptide
BSA	bovine serum albumin
CHD	coronary heart disease
CHF	congestive heart failure
CK	creatine phosphokinase
CRP	C-reactive protein
cTnI	cardiac troponin I
cTnT	cardiac troponin T
CV	cyclic voltammetry
CVDs	cardiovascular diseases
DNA	deoxyribonucleic acid
DPV	differential pulse voltammetric
EIS	electrochemical impedance spectroscopy
ELISA	enzyme-linked immunosorbent assay
ELONA	enzyme-linked oligonucleotide assay
FET	field-effect transistor
FICA	fluorescence immunochromatographic assay
GDF15	growth differentiation factor 15
GDF8	growth differentiation factor 8, myostatin
HF	heart failure
hFABP	heart-type fatty acid-binding protein
HSA	human serum albumin
IgE	immunoglobulin E
IL-6	interleukin-6
LOD	limit of detection
MR-proADM	mid-region-proADM
Nt	nucleotides
NT-proBNP	N-terminal proBNP
POC	point-of-care
RNA	ribonucleic acid
SELEX	systematic evolution of ligands by exponential enrichment
SERS	surface-enhanced Raman spectroscopy
SOMAmer	slow off-rate modified aptamers
SPR	surface plasmon resonance
sST2	soluble suppression of tumorigenicity 2
ST2	suppression of tumorigenicity 2
ST2L	transmembrane suppression of tumorigenicity 2
SWV	square wave voltammetry
TGF-β	transforming growth factor-β
TNFα	tumor necrosis factor alpha
WHO	world health organization
